# Philadelphia Medical Society

**Published:** 1841-12-18

**Authors:** 


### PROCEEDINGS OF SOCIETIES.

### PHILADELPHIA MEDICAL SOCIETY.

*Session of Nov. 27.*

LECTURE OF DR. R. COATES—CONCLUDED.

Having repudiated the attempt to connect the nervous phenomena forming the subject of the lecture with the action of any known agent in imponderable chemistry, and having disproved, to his own satisfaction, their necessary connection with the mind of the patient, Dr. Coates endeavoured to free the subject from the mass of physical and metaphysical nonsense with which it has been loaded by charlatanry and imaginative assumption, in order to point out the rational route of investigation in researches into the cause or history of those phenomena.

The cause being, according to his argument, *purely physical*, and acting *solely on the nervous system*, the results cannot by possibility consist in any thing else than the diminution, elevation, modification, or transfer of the natural functions of the nervous fibres, or the establishment of nervous functions altogether novel in character. He proceeded to comment upon the natural displays of most of these changes, in diseases affecting the nervous system of animal life; as the peculiar relations between the fibres of this system and consciousness and will, (which powers Dr. Coates regards—not as functions of the nerves or brain, but as purely psycological attributes, *merely awakened into action* by the organic structure of animals,) enable us to study their changes through their secondary mental consequences.

With regard to elevations and depressions of nervous functions by disease, illustrations were adduced from the intense susceptibility of the eye and ear in phrenitis, &c., and the loss of their functional power in many morbid conditions of the frame, unattended with any structural or local change in those organs, as in symptomatic amaurosis, &c.

Both these states were witnessed, says the lecturer, in the case of the literary gentleman subjected to mesmeric action, whose case has been already mentioned. In this case the acuteness of hearing was duplicated, and the sense of sight apparently lost. This elevation or depression was considered by the lecturer as a sufficient explanation of many of the phenomena of the mesmeric catalepsy and hysteria.

Modifications of the functions of the nerves, he remarked, are also common—as is evidenced by the phenomena of dreams, visions, and optical illusions, in which the mind receives impressions of form, colour, and other properties of matter, whether the object has a real existence or not, and this on account of the operation of agents upon the nervous fibres, which, in their normal condition, could not awaken any such perceptions. Colour may be perceived when no light reaches the eye; vivid and apparently real objects may be impressed on the mind when no such object was present. From these facts, the lecturer argued that other than the natural stimuli may act on a nerve, when in a condition modified by disease, in such a way as to produce on the mind the effects of the natural stimulus, and hence, that it would not be entirely out of the order of nature that light should awaken hearing, and the waves of sound, the sense of sight—as pressure on the eye is well known to do. He contended that there was nothing in the structure of the eye absolutely essential to sight; for that insects evidently saw a single object just as distinctly with eyes that repeated the image many hundred times, as man with only a single image on each eye; and many of the inferior animals, such as certain worms and moluscæ, evidently possess vision or its equivalent, without any thing worthy the name of eye—the part of the surface in which the power resides being covered with cuticle, or a modification of cuticle, without any subjacent structure capable of receiving or forming an image at all—a fact well known to all comparative anatomists. The lecturer saw, therefore, nothing contrary to the usual order of nature in the supposition that animals might see by means of the skin.

Granting the truth of the doctrine that the nervous system of one individual may exert a physical influence over that of another individual, this modification of function in the nervous system might account for the apparent community of senses between the mesmeric subject and the operator; for the influence of the nervous system of the latter exerted upon the nerves of the former, already modified by disease, might awaken in his mind the hallucination of the sense of taste, smell, or vision, without carrying us beyond the bounds of physical or physiological probability: and here it is proper to remark that the lecturer regards physiology as a purely physical science; denying that consciousness, will, or any veritably mental phenomenon, is essentially the result of the organization of an animal;—being a pure immaterialist in metaphysical faith.

On the subject of transfers of function from one nerve to another, Dr. C. dilated on the vicarious actions of one organ for another. The skin takes on, he thinks, an increased respiratory office in cases of diseased lungs;—it probably acts for the liver in jaundice;—it secretes stercoraceous effluvia in the fetid sudor of strangulated hernia;—it discharges urinous matter in some diseased conditions of the kidneys.

The universality of ossification, and the history of fractures, show that all the organs, and the common cellular tissues are capable of secreting bone, &c. Whence he arrives at the conclusion that the law of vicarious action is universal among the organs, though the action is always modified by the peculiarities of the structure in such cases. But the vicarious action is more facile and perfect when the organ which is morbidly charged with the function very nearly resembles in structure that by which it is performed in health; as when the skin takes on the action of the abdominal or thoracic mucous membrane. The mutual resemblance in structure of the various nerves would lead us to conclude, *a priori*, that one nerve might readily enact the part of another, if we were careful to separate the truly nervous functions from *the mental phenomena of sensation*, so universally confused with them in physiological reasonings.

This vicarious action, he says, may account for several phenomena of the mesmeric state, and he once held the opinion that it might explain many asserted facts which he now refers to the peculiar sense usually, but perhaps unfortunately, termed *clairvoyance*.

Clairvoyance, the lecturer contends, is not *sight*, but a peculiar sense existing in all animals, but commonly escaping attention, because, *in man*, and *during health*, it exists only in that faint degree which, to adopt the language of comparative anatomists, may be termed *typical*. Children have it more perfectly than adults—savages, more marked than the civilized man—and the congenitally blind, far more conspicuously than those who enjoy vision. But, like all other senses, it may be almost indefinitely elevated by disease. It is this which gives to many individuals that clear perception of an approach to an obstacle in perfect darkness which many attribute to the reaction of air, or the existence of an atmosphere around solids, but which enables many to recognise a vacuity as well as a solid obstacle. To study the sense which perceives objects without contact, it is necessary to observe its display in those inferior animals in whom it exists—not typically but in high perfection. The horse, in the most misty and darkest night, trembles and obstinately refuses to proceed on approaching a precipice. The bat, with its eyes extinguished, flies round an apartment full of obstacles without touching any of them, choses his perch, and throws his claw upon it with great certainty, if Spallanzini is to be believed.

The bees wander through forests for miles, by the most devious course, and return, when loaded, in a straight line to the hive. Even when made to travel during the night, and labour during the day, on their way to more flowery regions, as is the custom in some parts of Holland and Belgium, the change of scene does not confuse them; they return in a "bee line" before nightfall, very few being lost. The cat, carried for miles from home in a sac, usually returns very directly to the kitchen, and the pig, under the same circumstances, takes the most direct practicable route to the sty, sometimes even sacrificing its life in the attempt to swim rivers for this purpose. But of all the examples, the strongest is furnished by the carrier pidgeon, which, when taken hundreds of miles from home, returns direct to its nest, though a large mass of the earth's rotundity intervenes between it and the object of its search, and no elevation capable of sustaining wing or life can enable it to employ the sense of vision as a guide. These phenomena are usually attributed to "instinct;" but Dr. C. considered it impossible to give any definition of instinct which would in any degree explain them: he regarded the attempt as a most miserable apology for ignorance, and believed them all to result from the existence of a peculiar sense, by the aid of which the nerves are physically impressed by external things without contact, and at indefinite distances.

He next mentioned several individuals in Philadelphia,—himself among the number,—who in youth or manhood enjoyed this sense in a degree somewhat wonderful,—which he declared to be not more remarkable than that some persons could exercise the attollentes and retrahentes auris muscles, though others could not: also, cases of rare indiosyncracy, in which the power existed in astonishing perfection, and among these was the celebrated instance of the old man who was stationed on the Signal Hill at the Mauritius and foretold the arrival of vessels, *regularly and surely for years*, when they were distant more than a hundred miles from the island: a fact not explicable by the existence of a mirage, which is never permanent and dependable. The lecturer then referred to several cases in which the sense was plainly and cleary developed under his own observation in somnambulism and natural hysteria, as well as in those upon whom he had operated himself in the mesmeric state—and explained the cause of the frequent apparent errors in the perceptions of the clairvoyant, by the imperfection of the developement of the sense and its consequent vagueness in those cases: stating that it was as reasonable to expect a man to see objects distinctly and describe them accurately, with eyes obscured by nebulæ, as to ask such results from those in whom the clairvoyance was not sufficiently developed.

He next proceeded to disprove many of the hypothetical notions which have induced observers to mistake the meaning of the phenomena actually exhibited in the mesmeric catalepsy and hysteria, by leading them to observe through a false medium of prejudice.

The presence of metals cannot interfere with the mesmeric condition, he remarked, in virtue of their chemical properties; for Mesmer himself produced his results by means of metallic combinations. Metals or magnets cannot be necessary to the results, for when the tractors of Perkins were cast aside, the effects of manipulation were unaltered. The mind of the patient is not under the *government* of the will of the operator, though generally *influenced by it*, doubtless through its reaction on the nervous system of the operator; for the patients often refuse to obey his orders, and, in clairvoyance, will frequently describe things unintended by him or unknown to him, and this in direct defiance of his stubborn purpose; as happened several times in the course of the experiments of the operator. Some patients will converse indifferently with any one present, others only with the operator, others again only with persons "placed in connection" by joining hands or with those who touch the operator. By a finesse, some of the latter class can be made to refuse to speak with one "placed in connection," and to reply only to some other individual near him. In fine, from his own experiments, and the verbal report of a friend on those of Professor Horner, Dr. C. is convinced that the subject of experiment is cognizant of all that passes, but *generally* converses only with the one to whom the attention is most strongly directed; because the sense of clairvoyance is often as distinctly under the command of the proper will of the patient, as far as the direction of attention is concerned, as is the sense of sight in a healthy man. The motions performed under the unexpressed will of the operator, (of the existence of which there can be no doubt,) appear to be purely involuntary and abstractly physical. Manipulation is not essential to the production of the mesmeric condition, for it often occurs without such intervention. Why it should be promoted by such ridiculous means is inexplicable, unless on the supposition that, by action upon the mind of the patient, it produces a reaction on the nervous system, disturbing it and thus rendering it more susceptible of the morbid change upon which depends the modification of its functions.

It is to be regretted that our limited space, and the practical character of this Journal, will not allow us to do the lecturer justice by quoting the many curious facts by which he supported his peculiar views. The length of the lecture and other circumstances precluded the possibility of much debate, and we therefore omit the remarks by Dr. B. H. Coates, Prof. Huston, and Dr. Wiltbank, trusting that before the season closes the subject of the functions of the nervous system will become the subject of further comment. We close this article with the concluding paragraphs of the lecture.

"All the phenomena of the seeming government of the will, really result from the physical impressions directing or enticing the attention of the patient through the medium of the nervous system of the operator—itself modified, it may be, by the reaction of his mind.—But the impressions conveyed through the sense of clairvoyance are received by the mind of the subject in the same manner as the impressions on the sense of tact. The mind of the patient, then, instead of being the primary link in the chain of causes which produce the mental phenomena observed in the mesmeric condition, miscalled sleep, as the metaphysical dreamers would have us believe, is, in reality, the very last link in the series.

"Believing that the very curious facts connected with the several trains of symptoms exhibited in Hysteria, Catalepsy, Somnambulism, and the result of what is termed by many Mesmerism, have hitherto stood in great degree isolated from the domain of science, and have furnished plausible foundation for many monstrous opinions at war with religion, morals, health and happiness. I have endeavoured this evening, to point out the only chain of concatenation by which they appear to me capable of scientific association.

"If my views be correct, there is nothing true in mesmerism which admits not of explanation upon principles parallel with those already established in Physiology—nothing miraculous—nothing more mysterious than is existence itself. But, perceiving, without the aid of more mental *clairvoyance* than should belong to every philosophical investigator, that the study of the mutual vital reactions of the nervous systems of different individuals is destined to give birth to a new and most important era in physical and moral science when we lie low, I was unwilling that the dread of persecution, or the *much more serious* danger of misconception, should deter me, as it has done many, from bearing testimony to the truth of valuable facts, and making the statement of legitimate conclusions, because the whole subject has been rendered demoralising by the infidel and ridiculous by the charlatan.

"Neither the anathemas of Popes, the condemnation of academies, nor the outcry of the ignorant, can render less honourable the calm pursuit of scientific truth. And in the defence of the opinions merely shadowed forth in the course of this ill digested lecture before this logical and reasoning tribunal,—Mr. President, and gentlemen of the Society!—There lies my glove."

